# The Nutraceutical Alliin From Garlic Is a Novel Substrate of the Essential Amino Acid Transporter LAT1 (SLC7A5)

**DOI:** 10.3389/fphar.2022.877576

**Published:** 2022-03-24

**Authors:** Raffaella Scanga, Mariafrancesca Scalise, Filomena Rovella, Teresa Maria Rosaria Regina, Michele Galluccio, Cesare Indiveri

**Affiliations:** ^1^ Unit of Biochemistry and Molecular Biotechnology, Department DiBEST (Biologia, Ecologia, Scienze Della Terra), University of Calabria, Arcavacata di Rende, Italy; ^2^ CNR Institute of Biomembranes, Bioenergetics and Molecular Biotechnologies (IBIOM), Bari, Italy

**Keywords:** alliin, garlic, nutraceutical, membrane transport, LAT1, SLC, liposomes

## Abstract

The plasma membrane transporter LAT1 (SLC7A5) is a crucial player for cell homeostasis because it is responsible for providing cells with essential amino acids and hormones. LAT1 forms a functional heterodimer with the cell surface antigen heavy chain CD98 (also known as 4F2hc and SLC3A2), a type II membrane glycoprotein, which is essential for LAT1 stability and localization to the plasma membrane. The relevance of LAT1 for human metabolism is also related to its altered expression in human diseases, such as cancer and diabetes. These features boosted research toward molecules that are able to interact with LAT1; in this respect, the recent resolution of the LAT1-CD98 3D structure by Cryo-EM has opened important perspectives in the study of the interaction with different molecules in order to identify new drugs to be used in therapy or new substrates of natural origin to be employed as adjuvants and food supplements. In this work, the interaction of LAT1 with alliin, a garlic derivative, has been investigated by using a combined approach of bioinformatics and *in vitro* transport assays. Alliin is a nutraceutical that has several beneficial effects on human health, such as antidiabetic, anticarcinogenic, antioxidant, and anti-inflammatory properties. The computational analysis suggested that alliin interacts with the substrate binding site of LAT1, to which alliin was docked. These data were then confirmed by the competitive type inhibition measured in proteoliposomes. Interestingly, in the same experimental model, alliin was also revealed to be a substrate of LAT1.

## Introduction

The human SLC7A5 transporter, also known as LAT1, belongs to the SLC7 family together with twelve other proteins. LAT1 forms a heterodimer with another protein belonging to the SLC3 family, namely, SLC3A2, known as CD98 (Mastroberardino et al., 1998; Fotiadis et al., 2013; Scalise et al., 2018). The interaction between the two proteins is stabilized by a covalent bond occurring at the level of two conserved cysteine residues, as previously demonstrated by the biochemical approaches and recently confirmed by the 3D structures solved in an inward open conformation (PDB: 6IRS, 6IRT, and 6JMQ) (Lee et al., 2019; Yan et al., 2019) and in an outward occluded conformation (PDS: 7DSK, 7DSL, 7DSN, and 7DSQ) (Yan et al., 2021). Different roles have been proposed for the two proteins: LAT1 is the transport competent subunit involved in mediating amino acid flux across the plasma membrane, whereas CD98 is an ancillary protein with no role in transport function (Napolitano et al., 2015). CD98 is a multiplayer protein with several functions other than the interaction with LAT1 or other transporters of the SLC7 family (Cantor and Ginsberg, 2012; Fotiadis et al., 2013); probably, CD98 is involved in routing LAT1 to the definitive location in the plasma membrane (Cormerais et al., 2016). In normal conditions, LAT1 is expressed in the placenta and the blood–brain barrier epithelia (Verrey et al., 2004; Fotiadis et al., 2013; Scalise et al., 2018). LAT1 has also been described at the lysosomal membrane of HeLa cells (Milkereit et al., 2015). The described distribution meets the biological role of LAT1 in human physiology, mediating the transport of the essential amino acids which are strongly required for normal brain development and fetus growth. In good agreement, KO embryos for murine LAT1 are not vital (Ohgaki et al., 2017) and the intrauterine growth restriction (IUGR) is characterized by low levels of LAT1 substrates in the placenta (Pantham et al., 2016). Over the years, the functional properties of LAT1 have been characterized in different experimental models, employing intact cells, xenopus oocytes, and recombinant protein reconstituted in proteoliposomes (Kanai et al., 1998; Mastroberardino et al., 1998; Napolitano et al., 2015). The studies revealed that LAT1 is a sodium-independent antiporter of essential amino acids; it obeys a random simultaneous kinetic mechanism and is positively regulated by physical interaction with cholesterol in the membrane and with intracellular ATP (Mastroberardino et al., 1998; Napolitano et al., 2017a; Cosco et al., 2020). Histidine is the favorite substrate and, physiologically, is preferentially exported from cells in antiport with other essential amino acids. This mechanism has been recently demonstrated in a study conducted on autism spectrum disorder (ASD)-related mutations of LAT1 employing both *in vivo* and *in vitro* models. In particular, mice harboring mutated LAT1 showed an accumulation of histidine in the brain and lower levels of the other LAT1 substrates (Tarlungeanu et al., 2016). An even greater scientific interest around LAT1 started when the protein was found over-expressed in virtually all human cancers that rely on essential amino acids for supporting their increased transport rate (Fuchs and Bode, 2005; Scalise et al., 2021). Moreover, leucine taken up *via* LAT1 also has regulatory function on other metabolic pathways, such as glutamine utilization in tricarboxylic acid (TCA) cycle that is a hallmark of cancer cells (Damiani et al., 2017; Scalise et al., 2017). Given the described background, it is not a surprise that LAT1 is considered a hot pharmacological target for drug design in the field of anticancer therapy; indeed, a large number of articles dealt with LAT1 inhibitors proposed as potential drugs for anticancer therapy [Scalise et al. (2021) and refs herein]. One of such compounds already reached the clinical trial for cancer treatment, that is, the tyrosine analog JPH203 (Okano et al., 2020). LAT1 is relevant to pharmacology not only as a drug target but also as a drug transporter. Indeed, great efforts have been made to design prodrugs, namely, molecules based on LAT1 accepted amino acid scaffolds conjugated with drugs able to cross virtually impermeable epithelial barriers, such as the blood–brain barrier (BBB). This approach is fundamental in the case of neurological disorders that are difficult to treat due to the low permeability of BBB to xenobiotics (del Amo et al., 2008). In this context, natural compounds constitute an enormous chance of identifying substrates and inhibitors of LAT1 with a potential application to human health. A particular class of natural compounds is nutraceuticals, a term derived from “nutrition” and “pharmaceutical” indicating a specific food (or a part of food) that gives benefits to health (Sanchez-Sanchez et al., 2020). One eminent example of nutraceutical is the group constituted by bioactive molecules extracted from garlic (*Allium sativum*). In particular, alliin is a water-soluble and odorless compound, reaching 1.8% of the total organosulfur compounds in garlic bulbs. Alliin is produced by the oxidation of S-allyl-glutamyl-cysteine and is the precursor of another common garlic compound named allicin (Amagase et al., 2001). The biological properties of alliin have been extensively studied in different models demonstrating that this compound has antidiabetic, anticarcinogenic, antioxidant, and anti-inflammatory effects (Chung, 2006; Quintero-Fabian et al., 2013; Zhai et al., 2018; Sanchez-Sanchez et al., 2020). Indeed, alliin is nowadays considered the most representative nutraceutical in garlic. Moving from these premises, we here describe the LAT1–alliin interaction using bioinformatics validated by *in vitro* transport assays.

## Materials and Methods

### Materials

*E. coli* Rosetta(DE3)pLysS cells were from Novagen (Rome, Italy); His Trap HP and PD10 columns were from GE Healthcare; L-[^3^H]histidine was from American Radiolabeled Chemicals (ARC Inc., United States); C_12_E_8_, Amberlite XAD-4, egg yolk phospholipids (3-sn-phosphatidylcholine from egg yolk), cholesterol, Sephadex G-75, imidazole, L-histidine, alliin, and all the other reagents were from Merck Italia (Italy).

### Over-Expression and Purification of Recombinant hLAT1 Protein

hLAT1 was over-expressed in *E. coli* Rosetta(DE3)pLysS and purified using affinity chromatography on ÄKTA Start, as previously described (Cosco et al., 2020). In brief, the supernatant from solubilized *E. coli* cell lysate was loaded on a His Trap HP column (5 mL Ni Sepharose) pre-equilibrated with 10 ml of a buffer composed of 20 mM Tris–HCl pH 8.0, 10% glycerol, 200 mM NaCl, and 0.1% sarkosyl. After sample loading, the column was washed with 10 ml of a washing buffer (buffer A) composed of 20 mM Tris–HCl pH 8.0, 10% glycerol, 200 mM NaCl, 0.1% DDM, and 3 mM DTE. The protein was eluted using 15 ml of a buffer (buffer B) composed of buffer A and 400 mM imidazole; fractions of 1 mL were eluted. Then, to remove imidazole and NaCl, 2 mL of purified hLAT1 was loaded onto a PD-10 desalting column equilibrated with a buffer composed of 20 mM Tris–HCl pH 8.0, 10% glycerol, 0.1% DDM, and 10 mM DTE; 2 mL of the desalted protein was collected for downstream functional assay.

### Liposome Preparation

For removing calcium phosphate from phospholipids, 3 mM EDTA was added to 10% egg yolk phospholipids and incubated for 15 min at RT. Then, chloroform was added at a 1:1 ratio with phospholipids, and the solution was centrifuged for 15 min at 12000 g, and at 4°C using a fixed angle rotor. The supernatant containing clean phospholipids in chloroform is evaporated by using a rotavapor at 40°C. Then, 7.5% of cholesterol was added to a phospholipid film obtained by the rotavapor and then was dissolved with chloroform. After the incubation under rotatory stirring (30°C 15 min 750 rpm), the solution was dried using the rotavapor. The lipid film was resuspended in 1.5 ml water (10% final concentration), and single bilayer liposomes were prepared by two sonication cycles of 1 min (1 pulse ON and 1 pulse OFF, 40 W) with a Vibracell VCX-130 Sonifier, as previously suggested (Mazza et al., 2021).

### Reconstitution of the hLAT1 Transporter Into Proteoliposomes

The desalted hLAT1 was reconstituted by removing the detergent from mixed micelles containing detergent, protein, and sonicated phospholipids by incubation with Amberlite XAD-4 in a batch-wise procedure, as previously described (Cosco et al., 2020). In brief, the mixture for reconstitution was composed of 7 µg purified protein, 100 µL of 10% C_12_E_8_, 100 µL of sonicated liposomes, 10 mM histidine (unless where differently specified in the figure legends), 10 mM DTE, and 20 mM Hepes Tris pH 7.0 in a final volume of 700 µL. Amberlite XAD-4 (0.5 g) was added to this mixture and incubated for 90 min at 1200 rpm on a thermoshaker incubator at 23°C.

### Transport Measurements

After the batch-wise procedure, 600 µL proteoliposomes were passed through a Sephadex G-75 column (0.7 cm diameter × 15 cm height) equilibrated with a buffer containing 20 mM Hepes Tris pH 7.0 and 10 mM sucrose to balance osmolarity. Then, the eluted proteoliposomes were divided into 100 µL aliquots for transport assay. For the uptake measurement, 5 µM [^3^H]-histidine was added to proteoliposome samples for starting the transport; the transport was stopped at desired times by adding 20 µM HgCl_2_ according to the stop inhibitor method as previously reported (Cosco et al., 2020). In the control sample, that is, blank, 20 µM HgCl_2_ was added at time zero. To remove the external (not taken up) radioactivity, 100 µL of each sample were passed through a Sephadex G-75 column (0.6 cm diameter×8 cm height). The samples were eluted with 1 ml 50 mM NaCl in 4 ml of scintillation mixture, and radioactivity was measured. The radioactivity in blank samples was used to subtract background radioactivity from the protein-associated one. The specific activity was calculated and expressed as nmol/mg at a given time or as nmol/mg/min in the case of transport rate measurement. For efflux measurements, proteoliposomes (600 µL), containing 10 mM histidine, were preloaded with radioactivity by transporter-mediated exchange equilibration by incubation with 5 µM [^3^H]-histidine for 45 min. External compounds were removed by another passage of the proteoliposomes through Sephadex G-75. Efflux measurement was started with or without adding the non-radioactive substrates histidine 100 µM or alliin 100 μM, to the preloaded proteoliposomes. The transport was stopped at desired times as previously reported.

### Computational Analysis

The three-dimensional coordinates of Cryo-EM LAT1 (PDB ID: 6IRT) (Yan et al., 2019) were downloaded, refined, and prepared within Glide from the Maestro suite v11.3 which consists of three steps: 1) preparation of the protein: addition of hydrogens, optimization of hydrogen bonds by flipping amino side chains, correction of charges, and minimization of the protein complex (Sastry et al., 2013). Default parameters were used. Chain A, corresponding to CD98 and all the ligands in 6IRT were removed; 2) preparation of the ligand using LigPrep: alliin was downloaded from PubChem in sdf format and the geometries were optimized assigning them in appropriate protonation states. Epik with default parameters were used; 3) preparation of the grid: receptor grids were generated keeping the default parameters of van der Waals scaling factor 1.00 and charge cutoff 0.25 subjected to OPLS3 force field. A cubic box of specific dimensions (30 × 30 × 30 Å) centered around selected residues (K204, F252, C335, S342, and C407) was generated for the protein.

### Induced Fit Docking Extra Precision and Visualization of Docking Results

Docking analysis was performed using the Glide SP protocol (Halgren et al., 2004). The number of poses generated was set to 20. Subsequently, a more advanced Glide function, namely, Induced Fit XP, was used on the best poses. This procedure allows the binding site residues to better adapt to the various poses of the ligand, resulting in a more optimized protein–ligand interaction. The workflow is composed of a job sequence in which ligand is docked with Glide, then Prime is used to refine and optimize side chains of all residues within 5.0 Å of ligand poses and, last, the ligands are redocked onto the relaxed receptor with Glide. The results yielded an IFD score for each exit pose (Friesner et al., 2004). Molecular graphics and visualization of docking results were performed with UCSF Chimera v.1.7 software (Resource for Biocomputing, Visualization, and Informatics, University of California, San Francisco, CA, United States).

### Data Analysis

Results are expressed as the mean ± SD. Grafit 5.0.13 software was used to calculate kinetic parameters, to derive percent of residual activity values in inhibition assays and to measure transport rate by the first-order rate equation.

### Other Methods

The amount of purified recombinant hLAT1 WT was estimated from Coomassie blue-stained 12% SDS–PAGE gels by using the Chemidoc imaging system equipped with Quantity One software (Bio-Rad), as previously described (Giancaspero et al., 2015).

## Results

### *In Silico* Prediction of Alliin Interaction With LAT1

**FIGURE 1:**
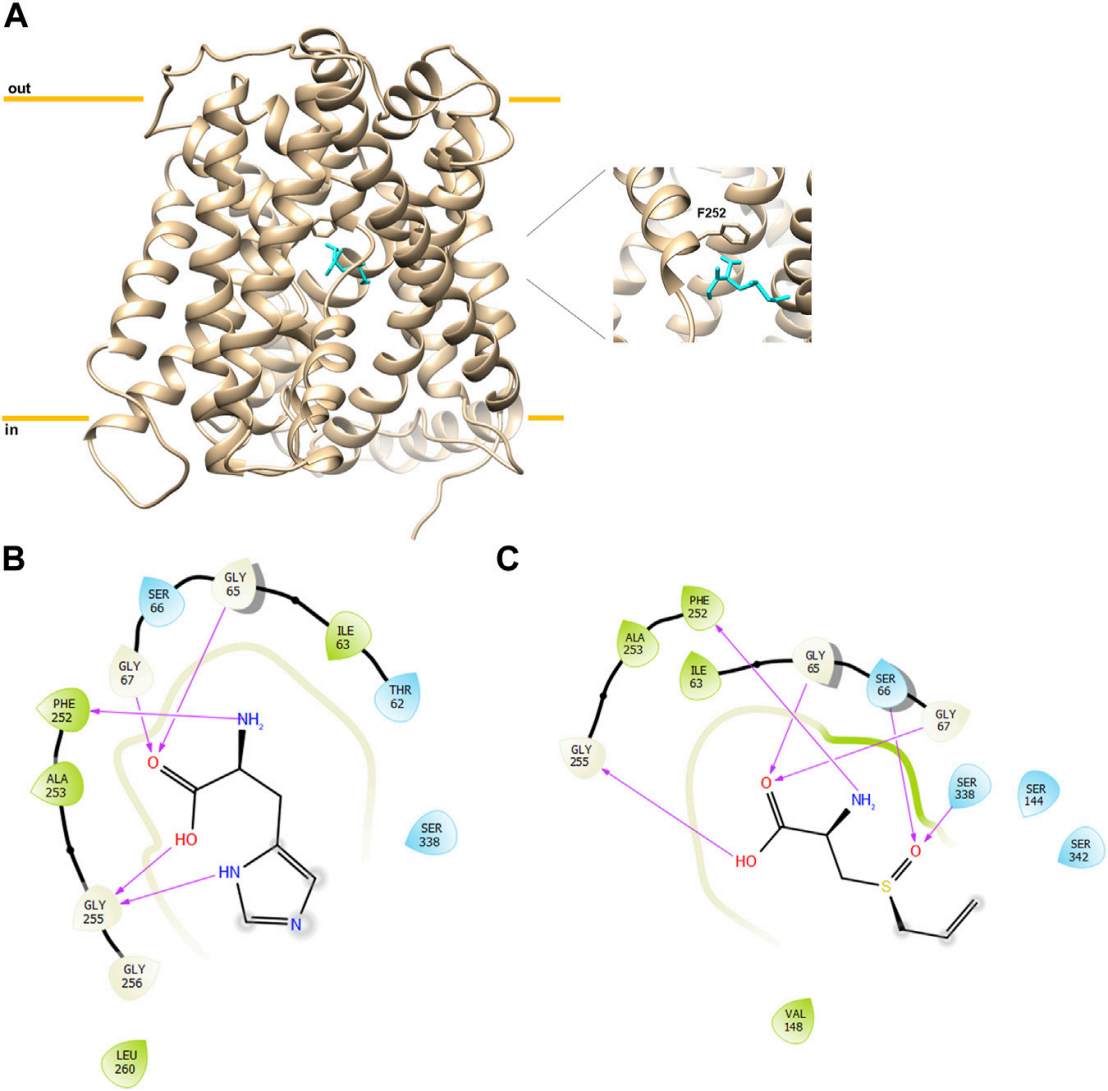
Docking analysis of hLAT1. The cryo-EM structure of hLAT1 in inward conformation (PDB ID: 6IRT, chain B) was represented as ribbon (sand) using Chimera v.1.7 software (https://www.cgl.ucsf.edu/chimera). In **(A)**, docking analysis was performed using InducedFit XP docking 5 as described in Materials and Methods. Molecular docking of alliin (sky blue) in the substrate binding site of hLAT1 with the gate residue F252 represented as sticks (sand). In the zoom, the pose of alliin with the docking score of −6.99 kcal/mol and a glide e-model of −72.237. The membrane and intracellular/extracellular environment are indicated. In **(B)**, 2D visualization of hLAT1 interaction with histidine. The arrows indicate the residues involved in the binding. In **(C)**, 2D visualization of hLAT1 interaction with alliin. The arrows indicated the residues involved in the binding.

The non-proteogenic amino acid alliin was selected as a potential LAT1 interactor, among the bioactive molecules of *Allium sativum* (garlic), considering its relative abundance in garlic extracts and its role in human pathophysiology. Alliin and other compounds, known to be present in *A. sativum* (Amagase et al., 2001), were subjected to molecular docking analysis using the 3D structure of LAT1 in the inward open conformation (PDB 6IRT). Resulting binding affinity values are reported in Table 1, and alliin exhibited the highest affinity, being more than double than the average affinity of the other docked compounds. In particular, the binding energies of alliin derivatives, allicin, diallyl sulfide, diallyl disulfide, and diallyl trisulfide decrease following the increase in structural differences from alliin (Table1). In good agreement, S-allyl-cysteine, which is the compound with the nearest structure to alliin, showed the closest binding energy, even though still much lower than alliin. As shown in Figure 1A, alliin docked to the substrate binding site of LAT1 with a measured binding energy of -6.99 kcal/mol; interestingly, this value is in the same order of magnitude campared to that of histidine under the same docking conditions (Figure 1B and Table 1). This prediction is in good agreement with the already available findings on the minimal requirement for a molecule to interact with LAT1, that is, the presence of the amino acidic function. Indeed, the NH_2_ group of alliin interacts with the gating residue F252 (Figure 1C).

**TABLE 1:** Binding affinities derived from docking analysis.

Compound	Glide g-score (Kcal/mol)	Glide e-model	CID (PubChem)	Structure
L-Histidine	−6.92	−60.042	6274	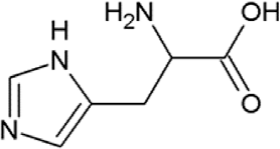
Alliin	−6.99	−72.237	87310	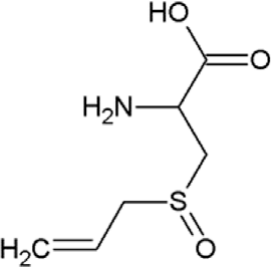
S-allyl-cysteine	−4.722	−44.606	9793905	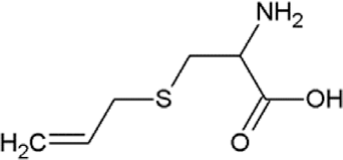
Allicin	−3.766	−33.58	65036	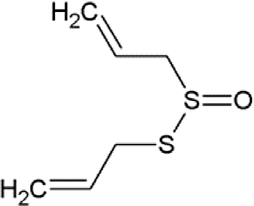
Diallyl sulfide	−1.116	−17.232	11617	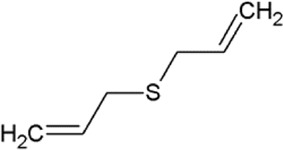
Diallyl disulfide	−2.066	−23.489	16590	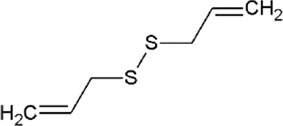
Diallyl trisulfide	0.041	−23.896	16315	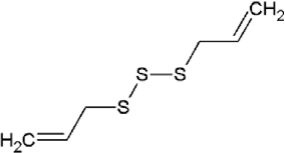

For each compound, the best pose has been identified by the lowest Glide e-model value (from Maestro suite); then, the binding affinities of the selected pose are expressed as Glide g-score (Kcal/mol). In the last column, the CID (compound identifier) was from PubChem database (https://pubchem.ncbi.nlm.nih.gov/).

### Kinetics of Alliin–LAT1 Interaction

**FIGURE 2:**
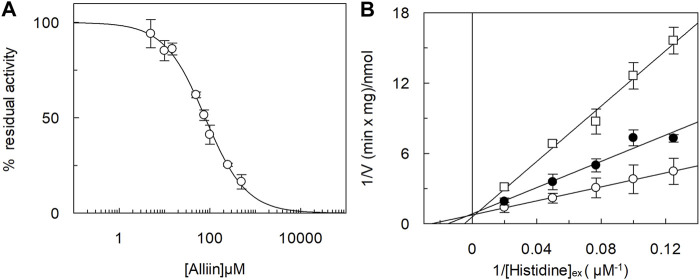
Inhibition analysis of the recombinant hLAT1 in proteoliposomes. The reconstitution was performed as described in Materials and methods. In **(A)**, dose–response analysis for the inhibition by alliin of the hLAT1. Transport was measured by adding 5 µM [^3^H]-histidine to proteoliposomes containing 10 mM histidine in the presence of indicated concentrations of alliin. Transport was stopped after 20 min as described in Materials and methods. Percent residual activity with respect to the control (without additions) is reported. In **(B)**, kinetic analysis of the inhibition by alliin. Transport was measured by adding indicated concentrations of [^3^H]-histidine to proteoliposomes containing 10 mM histidine and stopping the reaction after 20 min; 75 µM (●) or 150 µM (□) alliin was added in comparison to samples without inhibitor (○).The data were plotted according to Lineweaver–Burk as reciprocal transport rate vs. reciprocal histidine concentration. Results are the mean ± S.D. from the three independent experiments.

Following *in silico* simulation, alliin was experimentally tested to evaluate the effect on the transport function of LAT1 measured as an uptake of [^3^H]-histidine in exchange with internal histidine in proteoliposomes harboring human LAT1. A dose–response analysis was conducted (Figure 2A), and a reproducible inhibition of LAT1 was observed with a measured IC50 of 79 ± 9 µM. Then, the dependence of the transport rate on external [^3^H]-histidine concentrations in the presence of two alliin concentrations was measured (Figure 2B). The tested alliin concentrations were close to the measured IC50 value. The data were plotted in double reciprocal plots, according to Lineweaver–Burk equation, and a pattern of straight lines intersecting on, or close to, the y-axis was derived (Figure 2B). This result is in line with a competitive type of inhibition; from the plotted data, the half saturation constant Ki was calculated, being 50 ± 19 µM. Remarkably, the derived Ki value was similar to the IC50, in further agreement with the competitive type of inhibition.

### Transport of Alliin in LAT1-Reconstituted Proteoliposomes

**FIGURE 3:**
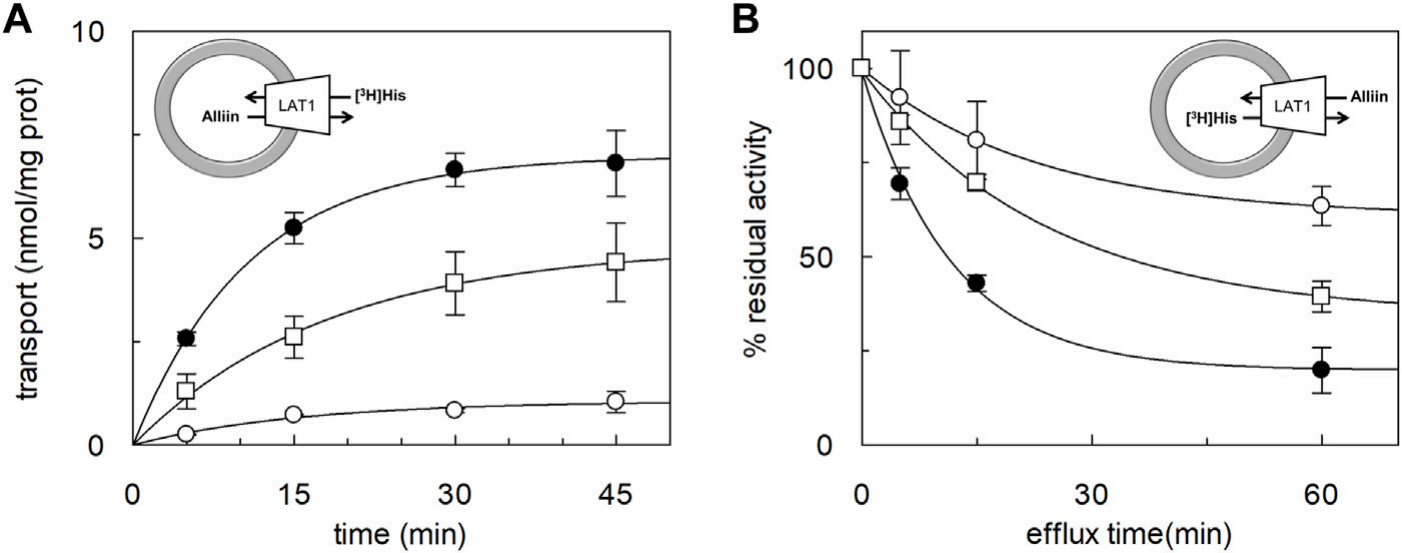
Time course of histidine uptake and efflux in proteoliposomes. The reconstitution was performed as described in Materials and methods. **(A)** Transport was started by adding 5 μM [^3^H]-histidine to proteoliposomes containing 1 mM histidine (●), or 1 mM alliin (□), or without the internal substrate (○). The transport reaction was stopped at the indicated times according to the stop inhibitor method as described in Materials and Methods. **(B)** Uptake of 5 μM [^3^H]-histidine was performed in 60 min. Then, the efflux of [^3^H]-histidine was measured in the absence of the external substrate (○), or in the presence of 0.1 mM of external histidine (●) or alliin 0.1 mM (□) at the indicated times. The data are calculated as the percent of residual activity with respect to the control sample (efflux time zero). Results are the mean ± SD of three independent experiments. The experimental model is depicted as a sketch.

The ability of alliin to act as a competitive inhibitor, together with the chemical features of alliin, that is, a sulfur-containing amino acid, drove us to investigate if alliin was, *per se*, a substrate of LAT1 in proteoliposomes. To address this issue, the peculiar transport cycle of LAT1 was exploited, that is, the obligatory exchange of the internal for external substrates (Figure 3, experimental sketches). First, the uptake of [^3^H]-histidine was measured in exchange for internal alliin. As a control, the canonical histidine_in_:[^3^H]-histidine_ex_ exchange was also measured (Figure 3A). The initial transport rate calculated for alliin was 0.25 ± 0.03 nmol*mg^−1^*min^−1^, only two times lower than that of histidine being 0.65 ± 0.05 nmol*mg^−1^*min^−1^. This result indicated that an exchange alliin_in_:[^3^H]-histidine_ex_ occurs, which is much higher than the control, that is, [^3^H]-histidine_ex_ uptake in the absence of the internal substrate. Thereafter, the symmetrical experiment was conducted, and the efflux of [^3^H]-histidine induced by alliin was measured (Figure 3B). In line with the data of Figure 3, alliin is able to stimulate the efflux of [^3^H]-histidine even though at a lower extent than histidine used at the same concentration. As a control, in the absence of external substrate, a negligible efflux of [^3^H]-histidine could be measured. These findings definitively demonstrated that an exchange [^3^H]-histidine_in_:alliin_ex_ occurs.

## Discussion

In this work, a novel finding, matching both the growing interests toward nutraceuticals and that toward membrane transporters, is described. Indeed, nutraceuticals need membrane transporters to be absorbed and distributed to human tissues. Moreover, membrane transporters are the first-level targets for interactions with drugs/nutrients that, on the one hand, might be source of toxicity, and, on the other, might be exploited for pharmacological effects. In this context, LAT1 plays crucial roles in human metabolism, and bioactive molecules contained in food represent a wide field of investigation. In this scenario, garlic (*Allium sativum*) has been recognized as a “super food” since more than 5000 years, and it has been used as a common remedy for several pathologies, including intestinal disorders, skin diseases, respiratory infections, and wound healing (Amagase et al., 2001; Majewski, 2014). Over the years, these observations, apparently derived from folklore, found scientific evidence, and garlic-derived compounds are listed as biomolecules with potential effects on cardiovascular diseases, immune system stimulation, detoxification from xenobiotics, and eventually cancers. For the sake of clarity, it has to be highlighted that some adverse reactions to garlic compounds exist (Amagase et al., 2001). Indeed, the quantity and quality of bioactive molecules contained in the commercial preparation of garlic are not always the same, triggering even opposite effects. From the literature data, it can be derived that alliin is the main bioactive molecule extracted from garlic, reaching 6–14 mg per gram of fresh garlic (Zhai et al., 2018). Alliin is a cysteine derivative, and when garlic tissue is damaged, it is rapidly converted to allicin, also known as garlicin, by the vacuolar enzyme alliinase. In contrast to alliin, allicin is oil-soluble and greatly unstable so that is considered virtually absent in commercial garlic products (Zhai et al., 2018). The systemic effects of alliin in ameliorating atherosclerosis, cardiovascular diseases, hyperlipidemia, hypertension, and various cancers have been documented in several studies (Amagase et al., 2001; Banerjee and Maulik, 2002; Rana et al., 2011). In this respect, LAT1 is a key player in human metabolism due to the mentioned ability of mediating the traffic of almost all essential amino acids; these features link LAT1 to several cell processes, ranging from protein synthesis to cell signaling functions (Mastroberardino et al., 1998; Scalise et al., 2021). These functions become even more relevant in those human pathologies with strong metabolic bases, such as cancer and diabetes. Indeed, in cancer, a rewire of cell metabolism occurs in which the substrates of LAT1 are required for several functions, such as the regulation of glutamate dehydrogenase by leucine, for the glutamine utilization in rewired cells (Fuchs and Bode, 2005; Damiani et al., 2017; Scalise et al., 2021). Moreover, in diabetes, a link between circulating BCAA and insulin resistance has been proposed (Lynch and Adams, 2014). Altogether these observations prompted us to evaluate the possible interaction between LAT1 and the garlic compound alliin. In this respect, we performed an approach based on *in silico* prediction, followed by experimental validation and molecular determination of the type of interaction between alliin and LAT1. Very interestingly, our *in silico* prediction showed that the best pose for alliin is in the same hydrophilic pocket that hosts the preferred LAT1 substrate histidine; in particular, the ammonium group of alliin interacts with the residue F252, the well-known gate for LAT1 (Napolitano et al., 2017a; Yan et al., 2019). The experimental data confirmed that alliin is able to inhibit the transport of histidine obeying a competitive-type of inhibition, as expected by the predicted interaction with the substrate binding site of LAT1. More importantly, we have shown that alliin is a substrate of LAT1 by exploiting our experimental tool of the reconstitution in proteoliposomes. Indeed, this methodology gives the advantage of performing transport assays without interferences deriving from the other membrane transporters and intracellular enzymes in intact cells (Scalise et al., 2013). This allows for precise determination of kinetic parameters and for discriminating between a pure competitive inhibition and an actual transport phenomenon. Interestingly, LAT1 revealed to be able to bidirectionally transport the garlic-derived amino acid alliin. This result is relevant for both theoretic biochemistry and applied physiology. In fact, the transport of alliin once more demonstrates that molecules harboring amino acid function are preferred interactors for LAT1, being in line with the published results on LAT1 inhibitors designed as substrate analogs (del Amo et al., 2008; Napolitano et al., 2017b; Okunushi et al., 2020). In terms of physiological relevance, the ability of LAT1 to recognize alliin as a substrate helps explaining the beneficial effects of alliin, whose distribution has never been deeply investigated. Furthermore, adding a natural compound to the list of LAT1 substrates may also be helpful for pharmacology considering that alliin could be even exploited as a Trojan horse to synthesize prodrugs for those epithelial barriers difficult to cross such as the BBB.

## Data Availability

The raw data supporting the conclusion of this article will be made available by the authors, without undue reservation.
